# Ten years on: a ‘*Montgomery* map’ for healthcare professionals in the United Kingdom and Beyond

**DOI:** 10.1093/medlaw/fwag012

**Published:** 2026-06-11

**Authors:** Rachael P Mulheron

**Affiliations:** School of Law, Queen Mary University of London, London, E1 4NS, United Kingdom

**Keywords:** *Bolam* test, informed consent, *Montgomery* duty, negligence, patient autonomy, tort

## Abstract

It has been a decade since the decision in *Montgomery v Lanarkshire Health Board* [2015] UKSC 11 was delivered, which definitively established a patient-focused test for deciding whether a healthcare professional (HCP) is under a duty to warn their patient of inherent risks. Given the significant cadre of cases since, it is an appropriate juncture to assess *Montgomery’s* implications for HCPs and for their advisers in two important respects, so as to develop a ‘*Montgomery map*’ or guide. First, in the ‘consultation phase’ between diagnosis and treatment—and in which the duty to warn is nestled—the *Bolam* test (as modified by the *Bolitho* ‘gloss’) remains the governing test of breach to a much greater extent than may have been anticipated when *Montgomery’s* patient-centric approach was affirmed, courtesy of *McCulloch v Forth Valley Health Board* [2023] UKSC 26. It is argued that *Montgomery* and *McCulloch* are entirely reconcilable within the nuanced landscape that the ‘consultation phase’ entails. Secondly, after a decade’s worth of case law, it is now possible to provide a detailed multi-factorial framework as to what, really, renders a risk so ‘material’ that it ought to be disclosed in order to comply with the *Montgomery* duty.

## I. Introduction

It is a decade since the Supreme Court of the United Kingdom (UKSC) changed the course of the law of negligence in respect of a healthcare professional’s (HCP) failure to warn a patient of an inherent risk^1^ associated with a medical treatment or procedure. The decision in *Montgomery v Lanarkshire Health Board* (‘*Montgomery*’)^2^ was very welcome and long overdue. Whether inherent risks should be disclosed to the patient was no longer to be determined by whether an HCP, in not disclosing them, acted in accordance with a practice accepted at the time as being proper by a responsible body of medical opinion (even though other HCPs might adopt a different practice about disclosure), per the ‘*Bolam* test’.^3^ Instead, the relevant test (‘the *Montgomery* Materiality Test’) asked which inherent risks the reasonable patient or the particular patient would consider to be important enough to have been disclosed by the HCP. Post-*Montgomery*, the latter has a duty:

to take reasonable care to ensure that the patient is aware of any material risks involved in any recommended treatment, and of any reasonable alternative or variant treatments. The test of materiality is whether, in the circumstances of the particular case, a reasonable person in the patient’s position would be likely to attach significance to the risk, or the doctor is or should reasonably be aware that the particular patient would be likely to attach significance to it.^4^

Hence, *Montgomery* placed patients’ decision-making at the heart of the consultative process, vindicated the importance of personal autonomy,^5^ and swept aside what must surely rank as one of the more opaque decisions of the House of Lords, three decades earlier, in *Sidaway v Board of Governors of the Bethlem Royal Hospital and the Maudsley Hospital* (‘*Sidaway*’) in which the majority had favoured the *Bolam* test with varying degrees of support.^6^ None of this was particularly novel—the Australian High Court had previously adopted a very similar analytical framework for failure-to-warn claims (and robustly declared its departure from *Sidaway*) in *Rogers v Whitaker*,^7^ and to which the UKSC in *Montgomery* paid careful attention.^8^ Not for the first time, a ground-breaking decision in UK law took account of, and refined, legal principles from elsewhere.^9^

What a welcome redirection and clarification that *Montgomery* provided, for in between *Sidaway* (1985) and *Montgomery* (2015), UK case law on the failure-to-warn allegation had become inconsistent, fragmented, and difficult to reconcile.^10^ Moreover, the choice of test, as between a ‘reasonable patient’ and a *Bolam*-driven assessment, was never about mere academic niceties. They could yield different results, such that considerable damages—or none at all—could turn upon whichever test was applied. The facts of *Montgomery* itself demonstrated this.^11^ The inherent risk of shoulder dystocia associated with a natural vaginal delivery (NVD)—already in the order of 9–10 per cent in Mrs Montgomery’s case because she was diabetic (and hence likely to have a larger baby) and small in stature, and coupled with a risk of permanent injury to the baby amounting to <0.1–0.2 per cent—did not require disclosure by the defendant obstetrician according to the *Bolam* evidence, for if customarily disclosed, it would make most women ‘simply request delivery by Caesarean section’ which carried its own risks.^12^ The reasonable patient test, which the UKSC preferred and applied, dictated quite the opposite conclusion: the severity of the hypoxic injury to baby Sam, and the sizable magnitude of risk to Mrs Montgomery’s own health from shoulder dystocia, meant that she should have been advised of those risks.^13^

The Supreme Court said, in *Montgomery*, that cases are ‘fact sensitive’,^14^ and so they are. But when taken together, post-*Montgomery* case law has laid down a now-extensive body of jurisprudence which enables the purpose of this article to be met, namely, the development of a blueprint as to how HCPs should seek to ‘*Montgomery*-proof’ themselves. The objectives of the *Montgomery* Map set out in this article are threefold (and undoubtedly overlapping)—that (1) patients are properly advised at law, (2) the human right of each patient to determine for themselves whether or not to proceed with treatment is respected, and (3) distressing (and expensive) litigation between patient and HCP might be avoided. The article seeks to achieve this in two parts.

First, it would be a grave mistake to consider that risk disclosure occurs in a ‘*Bolam*-free’ landscape. Post-*Montgomery*, jurisprudence shows that the advisory role of an HCP between diagnosis and treatment—the ‘consultation phase’—is constituted by several stages where *Bolam* firmly remains the governing test of breach. It is important that, a decade on, HCPs and their legal advisors appreciate when, precisely, the *Montgomery* patient-centric focus legally gives way to what peer professional opinion supports as reasonable practice. That nuanced landscape is analysed in Section II.

Secondly, the article contains the first exhaustive analysis in which every case in the last decade to have raised the failure-to-warn allegation for *ratio* consideration is examined so as to produce a framework that demonstrates, precisely, how courts have applied the *Montgomery* Materiality Test. As a result, it is now possible to set out the issues which have truly mattered at law because a reasonable patient, or the particular patient, would have regarded the undisclosed risk as being significant. Indeed, it is evident that those ‘two patients’ have been conflated in English jurisprudence since *Montgomery*, with no clear demarcation being maintained between them. By reference to relevant case law, Section III analyses the Materiality Test, and then promulgates (per Appendix A) a Guidance Note which aims to provide tangible and constructive assistance to HCPs and their legal advisers, to medico-legal organizations,^15^ and to courts which are called upon to adjudicate *Montgomery*-driven disputes. Significantly, the Materiality Test, which was articulated in the context of the birth of Mrs Montgomery’s child in 1999, is retrospective in nature, at least covering medical conduct from the 1990s (but perhaps not earlier than that).^16^ As Jay J remarked in *Tasmin v Barts Health*,^17^ it is a ‘fiction of the common law that [*Montgomery*] obligations existed in 2001 even if the relevant authority was not promulgated until much later’. There is nothing that HCPs can do to alter their historical conduct to ensure ‘*Montgomery*-compliance’, but guidance can surely assist for present and future cases.

While the focus is necessarily on UK case law, it is envisaged that for all those common law jurisdictions in which *Montgomery* jurisprudence is persuasive, from Hong Kong^18^ to Fiji^19^ and from Kenya^20^ to Singapore,^21^ the suggested *Montgomery* map set out herein will also be useful and instructive.

In *Montgomery*, the Supreme Court reiterated^22^ a retreat from the concept of medical paternalism, which, at times, had infused the judicial treatment of failure-to-warn claims. Recently, Turner J put the position well when he wrote that:

*Montgomery* marks, if not the final destination, at least the most recent resting point on a long journey away from a predominantly paternalistic approach to the relationship between doctor and patient as evidenced by the opinion of the majority in the House of Lords’ decision in *Sidaway*.^23^

This article seeks to contribute to this ‘long journey’ by marking *Montgomery’*s 10^th^ anniversary with clear, relevant, and helpful legal analysis, so as to translate the decision’s ratio into legal guidance as to what *really* matters ‘at the coal-face’ of medical services delivery.

## II. Why *Bolam* still retains great significance within the ‘consultation phase’

### A. The four stages of the ‘consultation phase’

The ‘consultation phase’ is positioned between two points—the patient’s *diagnosis* to begin with, and the *treatment/referral* that ultimately results. Any allegation of wrongdoing against an HCP in respect of either of these start-and-end points has long been governed by the *Bolam* test, and (since 1998) by the ‘*Bolitho* gloss’.^24^ By virtue of that caveat, *Bolam* evidence can be disregarded if it is considered by the court to be illogical, irrational, and indefensible—and that is the *sole* basis upon which the court can substitute its own view for that of the peer professional opinion which the defendant HCP adduced.^25^ Even a superiority analysis, whereby the court can simply prefer the expert opinion adduced by the injured patient, has been decried as being inappropriate for negligence claims arising out of diagnosis and treatment.^26^

As a patient navigates through the consultation phase between diagnosis and treatment, it is suggested that the HCP’s different responsibilities may be conveniently divided into four stages: devising a treatment plan; identifying and quantifying the risks associated with each treatment plan; disclosing material risks to the patient; and withholding risk information. For each of these stages, and for the purposes of developing a *Montgomery* map, it is important to delineate, precisely, just when a responsible body of peer professional opinion per the *Bolam/Bolitho* test is legally relevant in defending an allegation of wrongdoing made against an HCP, and when the question of breach is removed from the ambit of *Bolam* and is vested solely in the court. As one commentator has rightly noted, ‘[t]he setting of that boundary has important implications for practitioners, whether acting for patients or clinicians, as that boundary defines the issues on which expert evidence will be crucial.’^27^ The legal analysis of the consultation phase has been clarified (some judges have said ‘changed’^28^) since *Montgomery*, courtesy of the Court of Appeal’s decision in *Duce v Worcestershire Acute Hospitals NHS Trust* (‘*Duce*’)^29^ and the UKSC’s in *McCulloch v Forth Valley Health Board (Scotland)* (‘*McCulloch*’).^30^ In a nutshell:

#### 1. Stage 1: Devising the treatment plan

Where the treatment which is pursued by an HCP for a patient does not achieve the outcome which was hoped for (but where no negligence can be pointed to in the treatment itself), then the twin allegations of failure-to-warn-of-significant-risks and failure-to-advise-of-alternative-treatment-options are increasingly forming the basis of an overarching ‘lack of informed consent’ claim, post-*Montgomery*.^31^ If successful, the patient’s consent to the treatment is not vitiated to the extent that it is nullified, and indeed, that consent is sufficient to forestall any action in battery arising against the HCP—but an action in negligence will potentially be possible.^32^ Indeed, these twin allegations were actually part of Mrs Montgomery’s case (namely, that she was not warned of the risks of shoulder dystocia; nor advised of the alternative of an elective Caesarean section), but it was the former claim which was the clear focus of that UKSC judgment, leaving the latter relatively under-discussed.^33^ However, 8 years later, that alternative claim was brought to the fore in *McCulloch*, where Mr McCulloch, 39, died of pericarditis (inflammation of the pericardium, the sac surrounding the heart) 4 days after having been hospitalized with chest pains. The defendant cardiologist did not consider that Mr McCulloch’s symptoms, presentation, or the results of the ECG test fit a diagnosis of pericarditis, and he was discharged home with a prescription for antibiotics. It was alleged that an alternative treatment plan should have been offered, namely, non-steroidal anti-inflammatory drugs (NSAIDs), but this claim failed. In so deciding, *McCulloch* clarified several crucial legal principles about Stage 1 of the ‘consultation phase’.

First, when HCPs are evaluating the menu of all *possible* treatments for their patients, that is a matter of clinical judgment to which the *Bolam* test applies^34^—and if a treatment option was not known to a reasonably competent HCP at that time, there can be no breach by failing to inform the patient about it.^35^ Secondly, HCPs do not have to present *all* such options to the patient, but only those which are ‘clinically appropriate’ (ie, the ‘reasonable’) options^36^ (including those that may not be preferred by the HCP, for *McCulloch* does not give an HCP *carte blanche* only to put forward his preferred treatment option^37^). When assessing between the clinically non-viable (non-disclosable) and the clinically suitable (disclosable) treatment options, the *Bolam* test again applies.^38^ In *McCulloch* itself, the view that the prescription of an NSAID was not a clinically viable treatment option for Mr McCulloch was supported by *Bolam* evidence^39^ (and survived *Bolitho* scrutiny^40^), given that he was not in pain and there was no clear diagnosis of pericarditis. Post-*McCulloch*, however, several cases have demonstrated that, per the *Bolam/Bolitho* framework, the failure to put reasonable treatment options to the patient *did* constitute a breach.^41^ Thirdly, it explicitly follows, post-*McCulloch*, that failure-to-warn-of-material-risks and failure-to-advise-of-treatment-options may potentially arise out of the same ‘surgery gone wrong’, but they are to be determined by different legal tests. The former is governed by *Montgomery*/court review, and the latter is governed by the *Bolam/Bolitho* framework. In *Bilal v St George’s University Hospital*,^42^ Nicola Davies LJ put the position succinctly: ‘it is for the doctor to assess what the reasonable alternatives are; it is for the court to judge the materiality of the risk inherent in any proposed treatment, applying the test of whether a reasonable person in the patient’s position would be likely to attach significance to the risk.’

#### 2. Stage 2: Identifying and quantifying risk

Identifying which particular risks are associated with a proposed treatment plan (or of any reasonable alternative/s)—and assessing the magnitude of the risk and how to describe it, that is, whether in percentage (eg, ‘1–15%’) or descriptor (eg, ‘uncommon’) terms—are also matters of purely clinical judgment to which the *Bolam/Bolitho* test applies. In *Duce*, Hamblen LJ reiterated *Montgomery*-stated law that ‘[w]hat risks associated with an operation were or should have been known to the medical professional in question … is a matter falling within the expertise of medical professionals.’^43^ This means that, if the risk was not one which was known (or which was expected to be known) to apply to the surgery in question according to *Bolam* evidence (and, in *Duce*, the risk of ‘chronic post surgical pain’ arising from a total abdominal hysterectomy was not known in 2008 when the surgery was performed^44^), then the question of whether the patient should have been warned about it, per the *Montgomery* Materiality Test, never arises.^45^

#### 3. Stage 3: Disclosing risk information

Although *Montgomery* may have authoritatively held that what should be disclosed to a patient by way of risks is no longer determined by the *Bolam/Bolitho* test, it would be a legal error to consider such evidence to be *irrelevant*. *Montgomery* may have ‘ended *Bolam’s* reign’,^46^ but it has not removed its presence. One of the most notable features of Australian jurisprudence, following the very similar test espoused in *Rogers v Whitaker* in 1992, was how *Bolam* evidence retained significant relevance in failure-to-warn suits.^47^ Comments in *Duce* (that risks disclosure is ‘not something that can be determined by reference to expert evidence *alone*’^48^), and very recently in *Butler v Ward* (that ‘[e]xpert evidence may have a role to play in assisting the court’ in *Montgomery*-related litigation^49^)—together with a notation that it is ‘highly significant’ where the expert evidence on both sides agrees that a risk was material and should have been disclosed^50^—suggest that a similar pattern is unfolding in the UK too. Hence, *Bolam* evidence to the effect that there was a reasonable body of medical opinion which supported an HCP’s non-disclosure will be useful to gather as part of the defence to a failure-to-warn suit, *if* such evidence is procurable. It certainly cannot be determinative post-*Montgomery*, but it *may* nevertheless be relevant.

#### 4. Stage 4: Withholding risk information

Apart from the scenario in which the patient explicitly declares a wish not to be told of the risks,^51^ this stage invokes the defence of therapeutic privilege, the existence (but not the application) of which was recognized in *Montgomery* as a ‘limited exception’.^52^ It arises where the HCP ‘reasonably considers that its disclosure would be seriously detrimental to the patient’s health’.^53^ The defence lies beyond the scope of this article, given that its many unresolved issues, including the uncertain extent to which *Bolam* applies thereto (and which the author has explored in detail elsewhere^54^), were not the subject of any judicial discussion in *Montgomery*.

Of the stages within the ‘consultation phase’, it is Stage 1—the alternative treatment plan/s—which has attracted significant critical attention post-*Montgomery*, and that is examined next.

### B. Criticisms of *McCulloch* and responses thereto

The *McCulloch* approach, where an HCP only has to put *reasonable* treatment options to the patient, and that the *Bolam/Bolitho* test governs what a reasonable treatment option is, has been vehemently criticized in scholarly debate. For example, it has been said that: it returns the analysis to the paternalistic landscape that *Montgomery* was so keen to avoid^55^; it ‘mak[es] it harder for patients to argue they ought to have been offered an alternative treatment’^56^; it ‘significantly limits the application of *Montgomery* [if] patients are only told about the material risks of treatments that healthcare professionals deem reasonable’^57^; it ‘reintroduces the *Bolam* test by the back door [because] it is the medical profession which decides which option or options to present to the patient’^58^; it is problematical when *Bolitho* must be ‘relied upon to place limits on the unfettered discretion of clinical discretion in disclosing alternatives’^59^; and it ‘may not do justice to *Montgomery* and its avowed intent to champion patient autonomy’.^60^ One commentator despaired that, post-*McCulloch*, ‘[p]atients are now only entitled to exercise choices on the treatment options for their condition filtered by doctor and rubber stamped by the profession’.^61^ However, this author respectfully disagrees and considers that *McCulloch* embodies the correct legal approach regarding medical advice about treatment options for *two* reasons.

#### 1. Re-examining *Bolam*

Courtesy of *McCulloch* (and, to a lesser extent, *Duce*), what amounts to ‘clinical judgment’ throughout the consultation phase has been judicially delineated to a greater extent than occurred pre-*Montgomery—*and that brings to the fore the purpose and the limits of the *Bolam* test. When Stage 1 is viewed through that prism, then that provides the basis upon which *McCulloch* and *Montgomery* are properly reconcilable. Four points are pertinent in support of the author’s argument.

First, in the medical context, *Bolam* evidence is only required in a litigious dispute on matters that require ‘clinical judgment’, that is, matters where special skill or expertise is required, which the court itself does not possess and for which the court requires the assistance of expert evidence.^62^ Matters falling outside ‘clinical judgment’ are, properly, not governed by the *Bolam* test, and never have been.^63^ Hence, in the context under examination, it is for *clinical judgment* to assess whether, of the possible treatment options, the HCP’s evaluation of some of those is that they are hopeless, contraindicated, unlikely to achieve a better outcome, or even likely to inflict an adverse outcome. It is the *outcomes* of the ‘possibles’ which matter to that evaluation—and that is purely a matter for clinical judgment. Similarly, whether *all* possible treatments for the patient’s condition have been identified, identifying the risk/s associated with each ‘clinically-viable’ treatment option, and attributing to those options the risks as a percentage or as a descriptor of magnitude—all involve clinical judgment too. If an HCP is accused of any wrongdoing in respect of *any* of those tasks, the *Bolam/Bolitho* framework must be determinative. *McCulloch* simply confirms that, where a point of litigious dispute involves skill and competence which is beyond that possessed by the judge, it would be equally as inappropriate for the court to evaluate that question itself as to defer *holus bolus* to the views of an expert. Neither of these is the legal position in UK law. *McCulloch* prohibits the former, and *Bolitho* counters any overriding deference where the defendant HCP’s expert opinion as to what constitutes a clinically unviable treatment option is illogical and irrational.

Secondly, and speaking of *Bolitho*: in the author’s view, scholarly concerns^64^ about whether *Bolitho* scrutiny of the defendant HCP’s expert evidence is up to the task of rejecting any such evidence which opines, illogically and irrationally, that a treatment option was clinically unviable are overstated. The *Bolitho* test has been applied rigorously in the past where it has been needed^65^; and, post-*McCulloch*, it will properly focus attention more acutely on the need for the defendant HCP’s expert to address the alternative treatment options explicitly so as to divide them between the ‘clinically non-viable’ (non-disclosable) and the ‘clinically suitable’ (disclosable). There was something of a ‘gap’ in the *Bolam* evidence in *Montgomery* regarding the elective Caesarean section option and whether it was a reasonable alternative to an NVD,^66^ as the *McCulloch* Supreme Court^67^ and other scholars^68^ have noted. Going forward, any such gap *must* be addressed as part of the preparation of an HCP’s defence, and *McCulloch* appropriately emphasizes that.

Thirdly, information about risks and about treatment options does not have to be legally treated in the same manner. Quite the contrary. To remove from the ‘*Bolam* bucket’ an assessment as to whether a risk is material under the *Montgomery* Materiality Test is entirely consistent with other areas of clinical practice in which *Bolam* does not apply because there is no *clinical* judgment being exercised^69^; the court can (and should) make its own assessment of those matters. Whilst the author agrees with scholarly opinion that what amounts to *clinical* judgment can be blurred in some cases, and that the division, judicially-proposed, between ‘pure treatment’ and ‘pure diagnosis’ cases is not particularly helpful to that question,^70^ this does not detract from the issue at point, namely, that assessing treatment options, and dividing them between the clinically viable and the clinically inappropriate, categorically involves clinical judgment which belongs firmly within the ‘*Bolam* bucket’.

Finally, the *Bolam* test never requires a defendant HCP to anticipate the contrary view, to ‘second-guess’ what all peer professional opinion might think, and then conduct a cost–benefit analysis of the various viewpoints before coming to a decision, in order to preclude a finding of negligence. That, as a majority of the Court of Appeal said in *Adams v Rhymney Valley District Council*, would constitute an unacceptable rewriting of the *Bolam* principle and ‘would drastically alter the law of negligence’.^71^ Hence, it came as no surprise in *McCulloch* that, when it was submitted by the patient that an HCP should be under a duty to inform a patient of *all* treatment options that he knows (or should know) that a responsible body of medical opinion would regard as being reasonable, that submission categorically failed: it ‘would it render the law more difficult for a doctor to apply’.^72^ Not only that, but it would be *entirely inconsistent* with how the *Bolam* test has long been applied. It correctly follows that it is not relevant that all possible treatment options might be regarded as being *reasonable* by some responsible medical opinion; nor did it matter in *McCulloch* that (on the facts) there was a responsible body of medical opinion which supported the prescription of NSAIDs to Mr McCulloch. Each of those is entirely irrelevant to the way in which the *Bolam* test *actually* operates, where the HCP is exercising clinical judgment when assessing reasonable treatment options for the patient. Rather, it is sufficient that, where his clinical judgment is challenged to have been wrong and carelessly formed, the HCP is found to have acted in accordance with one body of peer professional opinion which is logical and rational. What some critics of *McCulloch* appear to advocate for is the introduction of a superiority test by which one body of expert evidence can be evaluated against the other when assessing what constitutes ‘clinically-viable’ treatment options—but appellate authority of the highest order has dictated against that path.^73^ Whether that view should be departed from is a question of an *entirely* different order (and falls well outside the scope of this article).

#### 2. Where would it lead?

The author’s second argument in support of the *McCulloch* analysis is this: suppose that, in *McCulloch*, the UKSC had decreed that *every* possible treatment option should be compelled by law to be put to the patient by an HCP. Quite apart from *practical* difficulties, namely, the descent into confusion that many consultations would demonstrate (as referenced in the judgment itself^74^), it would cause significant *legal* uncertainty.

For one thing, it would overturn a long-held line of authority, for suppose further that a patient was hellbent on following one of the contraindicated options. The English judicial position, both prior to^75^ and post^76^-*McCulloch*, is very clear: the relevant HCP is under no legal obligation to provide a treatment option that HCP believes to be clinically contraindicated (and which belief is supported by a body of responsible medical opinion). In that scenario, there is a (probable) legal duty on the HCP’s part to arrange a second opinion,^77^ but nothing more than that. In no way does English law (as some scholarly opinion has put it) ‘make the doctor the slave of the patient’s will’,^78^ or extend the notion of patient choice ‘to the right to demand treatment’,^79^ or require an HCP to present to a patient professionally unacceptable options.^80^ Quite simply, that has never been the law, and *McCulloch* affirmed that long-held position.

Moreover, if the insistent patient was somehow able to secure the contraindicated treatment option and it (unsurprisingly) resulted in some adverse outcome, then provided that the reasons for the contra-indication were adequately explained by the HCP to the patient, and the treatment was carried out with reasonable care and skill, where would the patient go, legally speaking? It is difficult to fathom what breach could be alleged against the HCP—and conversely, it is easy to fathom the causal difficulties and defences (contributory negligence, and even *volenti*) that might be asserted against the unhappy patient by the defendant HCP.

In summary, the correct balance was struck by *McCulloch* precisely because the role, scope, and application of the *Bolam* test were properly respected and adhered to. At Stage 1, the burden on the HCP is to use reasonable skill and care to distinguish between the clinically viable and clinically inappropriate treatments; and if it is alleged that the HCP got that assessment wrong, then, as a matter of clinical judgment, the *Bolam/Bolitho* test must be applied. Of course, re-invoking the relevance of peer professional opinion may be contrary to the autonomous and patient-centric ethos that *Montgomery* favoured—but when teased out in its four stages, it is plain that ‘clinical judgment’ pervades much of the consultation phase, and for which *Bolam/Bolitho* is rightly the appropriate governing test.

We now turn to the second part of the ‘*Montgomery* map’—assessing materiality of risk.

## III. Factors which indicate whether a risk is ‘material’

The *Montgomery* Materiality Test (reproduced earlier^81^) may purport to encompass two limbs—the reasonable patient in the patient’s shoes and the particular patient—but since *Montgomery*, the case law demonstrates that courts regularly conflate these two limbs (the objective and the subjective, respectively), maintaining no clear demarcation between them. The *procedure* probably bears closer analysis for the objective limb, whereas it is the *patient* that really matters for the latter.^82^ But even so, the whole ‘test of materiality is patient-centred’.^83^ It is certainly true that the greater the subjective factors that dictate materiality in any given case, the further the analysis departs from the ‘reasonable patient’,^84^ but that is how the analysis, post-*Montgomery*, has developed in reality. However, this author disagrees with the judicial view that the Materiality Test does not take account of the ‘irrational or wildly eccentric yet genuine’ patient.^85^ The second limb can do *precisely* that (and the similar limb in the *Rogers v Whitaker* test was taken to embrace such a patient, many years ago, in Australian jurisprudence^86^). Nevertheless, the *Montgomery* Materiality Test plainly does draw a distinction between objectively-significant and subjectively-significant risks. Thus, from the cadre of case law analysed for this article, the author has sought to divide the risks into precisely those two categories, both in the analysis which follows and in the Guidance Note contained in Appendix A, in order to provide as much assistance as possible to those whose professional and personal welfare are governed by the principles applicable in this difficult area of negligence law.

### A. A multi-factorial framework

In *Montgomery*, the UKSC put forward some material-risk factors in abstract terms^87^—but what follows seeks to flesh out, expand upon, and comprehensively analyse the multi-factorial framework which has emerged from the case law in the decade since *Montgomery*, so as to convert legal principle to practical guidance. Approximately 100 cases have been reviewed and inform the analysis which follows.^88^ The resulting multi-factorial analysis may seem lengthy, intimidating even–but provided that an HCP has these factors at the forefront of the mind when giving risk disclosure, case law suggests that material risks will have been addressed:

#### 1. The objectively significant risks identified post-*Montgomery*

##### a. The gravity of the injury, should the risk manifest

It is an obvious factor governing materiality, but it rarely operates on its own; its combination with the probability of the risk (the next factor) is all-important. Certainly, the mere existence of a serious injury has never ‘clinched’ the case, even in pre-*Montgomery* judgments which advocated for the ‘reasonable patient’ test. For example, in *Sidaway*,^89^ Lord Scarman did not consider that the risk of permanent paralysis should have been mentioned to elderly Mrs Sidaway; while in *Pearce v United Bristol Healthcare*,^90^ the risk of stillbirth was not material either, and where the risks in both cases were about 0.1 per cent. Still, the more serious the injury should the risk manifest, the more likely it is that a reasonable patient would wish to be informed of it. *Montgomery* itself demonstrates the factor, for both the gravity of shoulder dystocia to Mrs Montgomery, which itself constituted a ‘major obstetric emergency’, and the risk of brachial plexus injury to the baby, which could cause ‘severe injury such as cerebral palsy or death’, were material.^91^ Equally so, post-*Montgomery*, an undisclosed risk of damage to the patient’s optic nerve during an intraocular lens visual implant and which could severely restrict vision in that eye upon which the patient entirely relied for vision, should have been disclosed: ‘[t]he preservation of the functioning of her right eye was, understandably, of great importance to her’^92^; whilst the risk of paralysis as a result of spinal cord injury from a cervical discectomy fell into the same category.^93^ To reiterate, however, this factor is *never* sufficient on its own to require disclosure of the relevant risk.

##### b. Magnitude of the risk which manifested

*Montgomery* already instructs^94^ that materiality ‘cannot be reduced to percentages’^95^; and indeed, percentages may properly not be used at all, in favour of descriptors such as ‘very small’,^96^ ‘small’,^97^ or ‘rare’.^98^ Still, very low percentages will likely preclude materiality (a 1:100,000 risk fell into that category^99^), whereas anything in single-digit percentages is in real danger of being material.^100^ Within the range in between, the case law is (predictably) inconsistent. For example, a 0.1 percent risk of the grave consequence of paralysis was material^101^; but the same level of risk of permanent neurological damage as a result of hypoxic insult to a baby was not.^102^ Again, within that range, the factor is never conclusive, but interacts with others.

#### c. Whether an alternative (reasonable) treatment did not carry the same risk

As examined previously,^103^ an HCP is under a duty to disclose to a patient all *reasonable* treatment options that arise from a diagnosis of the patient’s condition. Assuming that both treatment options #1 and #2 are reasonable and hence disclosable, then where option #2 does not carry the same specific risk as does option #1, that specific risk arising from option #1 is likely to be considered material, for a reasonable patient would attach significance to its absence in respect of option #2. This point mattered enormously to materiality in *Diamond v Royal Devon and Exeter* (‘*Diamond*’),^104^ where the patient suffered from a post-operative abdominal hernia for which the defendant surgeon recommended and carried out a mesh repair. There was a risk that the presence of mesh could render a future pregnancy dangerous and contra-indicated, whereas a suture repair of the hernia, which was not discussed with the patient, was a reasonable option (albeit with a higher risk of failure), but did not carry the same risk should the patient become pregnant in the future. The risk of the mesh on the patient’s chances of giving birth successfully in the future was material. Of course, *Montgomery*^105^ falls within this category too—the option of an elective Caesarean section was reasonable and did not carry the inherent risk of shoulder dystocia, and hence, that risk associated with NVD should have been disclosed.

##### d. Where the procedure/treatment is novel and innovative

Where the procedure performed upon the patient amounts to a novel technique which is still in its infancy, and not well-established medically speaking, then its risks cannot be said to have been fully researched. The very fact of that novelty means that the risks associated with the procedure are ones which a reasonable patient would likely attach significance to and will be material. This factor was particularly evident in *Mills v Oxford University Hospitals*,^106^ where the patient had a brain tumour and underwent a minimally invasive endoscopically-assisted resection procedure with catastrophic results. His failure-to-warn lawsuit succeeded, partly because this type of resection ‘was a novel technique, still in its evolution’, and involved a smaller craniotomy, which did not give the neurosurgeon a direct line of sight, creating a risk of unseen damage to a blood vessel (which in fact happened).^107^ The previously mentioned factor was evident in this case too: a standard open craniotomy using a microscope would have eliminated the risk of unseen damage, and given that the latter was a reasonable treatment option employed by most neurosurgeons at the time, the inherent risk of blood vessel damage was material.^108^

##### e. Obvious risks, but of a very high magnitude

Any medical treatment is uncertain of success and may involve risk, even if the utmost skill and care are exercised—which is precisely why HCPs should never warrant an outcome by contract!^109^ The UKSC acknowledged in *Montgomery* that, so far as possible, adults should be capable of understanding that uncertainty.^110^ In *Sidaway*,^111^ Lord Templeman suggested that obvious inherent risks did not need to be warned about—and considered that the risk of damage to Mrs Sidaway’s spinal cord fell within that category. However, post-*Montgomery* case law has become more subtle than that. It suggests that obvious inherent risks which have a very high probability of occurring may be considered to be material—*notwithstanding* that they are obvious. Where, say, the risk of failure of a blood patch procedure to treat a spinal leak was reasonably high (50 per cent)^112^; or the one goal which the patient sought from eye surgery (to be able to read magazines again) had a very high risk of failure^113^; or the risk of pain following an operation was in the category of serious and permanent rather than transient and temporary,^114^ then all of these obvious risks should have been disclosed. It was their *magnitude*, rather than their obviousness, which rendered them material.

##### f. Purely elective surgery

Where purely elective surgery is proposed, it is easier to prove that non-disclosure of a risk was significant in the legal sense, because a reasonable patient would likely attach greater significance to risks that are purely a matter of his choice to undertake. The factor operates weakly, however. Post-*Montgomery* case law has certainly upheld that an undisclosed risk was material in the context of elective surgery: for example, the risk of permanent visual impairment arising from an elective lens implant^115^; and the risk of serious and permanent pain arising from an elective discectomy to resolve back pain.^116^ It was also significant in the pre-*Montgomery* case of *Chester v Afshar*,^117^ where the fact that the lumbar back surgery performed was elective rather than necessary assisted with a finding that the undisclosed risk of cauda equina syndrome was material.

##### g. What peer professional opinion considered to be material

As already discussed,^118^ it is strictly unnecessary for an HCP to anticipate the other viewpoint, and to consider which risks their peer professionals would consider to be material enough to be worthy of disclosure (what matters is whether the HCP’s non-disclosure is supported by a responsible professional body of medical opinion). However, case law suggests that an HCP ignores what his peers warn about at his peril. Certainly, it is no defence to materiality for the HCP to say that ‘I’ve never seen the risk manifest myself, in all my years of professional practice’. *Chester v Afshar*^119^ is a strong authority for that proposition. The defendant surgeon had conducted about 300 similar operations to Miss Chester’s per year, and over the span of 20–25 years had never seen a patient suffer from the inherent risk of cauda equina syndrome—yet none of that refuted the finding (at trial, and thereafter conceded on appeal) that the risk of cauda equina syndrome was material. But it goes further than that. If a HCP’s own experience is that, whilst he has not seen a manifestation of the risk, he is nevertheless aware that his peer group had experienced the contrary, then that can be an indicator of materiality, according to *Ollosson v Lee*.^120^ On that very basis, the risk of chronic pain following a vasectomy was a material risk (and it *was* disclosed).

##### h. Risk that the information which underpins a recommended treatment is incorrect, irrelevant, or both

There are still aspects of the duty-to-warn landscape that have yet to be judicially examined post-*Montgomery*. Yet, in an article of this sort, they cannot be ignored. Take, for example, clinical practice algorithms which may set out in ‘decision-making trees or flowcharts’ a variety of matters, say: a range of medical diagnoses or treatment options. Judicial warnings that algorithmic flowcharts do not define ‘risks’, let alone their materiality for the patient concerned, should be heeded.^121^ But more than that, such algorithms are ‘built upon’ machine-learning (ML) systems, which utilize data and assumptions, and which may be wrong or inapplicable in the particular patient’s case. That is an inherent risk, for no amount of reasonable care and skill on the HCP’s part can remove it. Should that risk which underlies ML-generated diagnoses or treatment options be disclosed? English case law has not yet had to grapple with this conundrum, but one example provided in scholarly literature^122^ illustrates: IBM developed a ML ‘decision support system’ called Watson,^123^ and developed Watson for Oncology. The data fed into the ML algorithm was gathered from a single cancer-treating hospital in New York. Oncologists in Taiwan noticed that Watson’s therapeutic recommendations of drug treatment for their cancer patients showed bias towards how oncology drugs are prescribed in the USA, in that Taiwanese patients are regularly prescribed lower doses of drugs to minimize side effects. Scholars have suggested that the risks posed by the data being used for a ML’s statistical model ‘[should] be regarded as being essential for the patient to make an informed decision’.^124^ This scenario raises questions as to the very definition of an ‘inherent risk’ and its materiality in the post-*Montgomery* landscape.

#### 2. The subjectively significant risks identified post-*Montgomery*

##### a. The patient’s employment

As part of the consultation phase, the patient’s employment should be ascertained, because that may render a risk material in the legal sense if its manifestation will hinder or preclude the patient from making a living in that chosen occupation or profession. Several post-*Montgomery* cases attest to this. For example, one patient should have been warned about the risk of lingual nerve and inferior alveolar nerve damage as a result of a lower wisdom tooth extraction, which could (and did) leave the patient with a significant speech impairment—a disastrous consequence, given her occupation as a speech and language therapist.^125^ Similarly, the risk of a ‘hoarse’ voice arising from injury to the laryngeal nerve from spinal surgery would be singularly important to a teacher (and that risk was rightly disclosed, given its materiality)^126^; and a claim was not summarily dismissed but sent for trial, where the patient alleged that, had he been warned of the risk of avulsion associated with a hip resurfacing procedure, he would have regarded the resultant hip weakness as being significant, for it prevented him from undertaking work as a ski instructor.^127^

##### b. The patient’s physical characteristics or existing medical condition

If *either* the patient’s physical characteristics (such as age, weight, or ethnicity) *or* some existing medical complaint from which the patient suffers elevates the probability of a risk occurring for that particular patient compared with a person from the general population, then that patient who is so afflicted would attach significance to it. Albeit that it is broadly cast, HCPs should be alive to anything that fits within this description, for it has mattered, legally, under the *Montgomery* Materiality Test. *Montgomery* itself is indicative of this factor, for the risk of shoulder dystocia associated with an NVD was elevated because of Mrs Montgomery’s small stature and her diabetic condition, which meant that she was likely to have a larger baby.^128^ Similarly, in *Keh v Homerton University Hospitals* (‘*Keh*’),^129^ the patient was at greater risk of emergency Caesarean section, given that she had a higher-than-average BMI, was a first-time mother, was in her early 40s, and black African women have a higher Caesarean rate than the general maternal population; whilst in *Negus v Guy’s and St Thomas* (‘*Negus*’),^130^ the patient was a small lady with a higher-than-recommended BMI, which rendered her at greater risk of needing aortic root enlargement because the intended aortic valve might not fit the aortic root during surgery. In both cases, the risks should have been disclosed (albeit that causation failed in both).

##### c. The patient’s medical history

Where some past medical episode in the patient’s history renders an inherent risk more likely to manifest for that patient compared with the general population, that is a relevant factor pointing to materiality. It may combine with the patient’s physical characteristics (per the previous factor), but even so, it can elevate the probability of risk for that particular patient and assume materiality for that very reason. For example, where a past Caesarean section put a mother at greater risk of uterine rupture during an NVD^131^; or where a patient was more at risk of sepsis following a ureteroscopy because of ‘her age, her sex, her [physical fitness] score, and *her history of urinary infection*’,^132^ then the respective risks were material and required disclosure. In the latter case, the patient died of multi-organ failure secondary to sepsis, where the risk of sepsis from ureteroscopy generally was in the region of 0.3 percent, but there was an increased risk of 0.6–0.7 percent for that particular patient. Interestingly, the warning of sepsis *was* given, not for the correct reason (the patient was told that a previous episode of post-operative urosepsis elevated her risk, but that was medically incorrect), but the wrong reason did not vitiate the defendant surgeon’s having met the *Montgomery* duty.^133^

##### d. The patient’s educational background

With education may come a greater appreciation of what a risk’s manifestation could mean. If a patient would (because of his medical knowledge) have appreciated the risks better than the general population, then there is case law support for the proposition that those risks are significant for that particular patient. The patients in both *Negus*^134^ and *Webster v Burton Hospitals*^135^ possessed nursing qualifications. In *Negus*, it was said that the patient ‘had been a nurse and had previously undergone a number of surgical procedures; she might reasonably have been seen as someone with a greater appreciation of the potential risks and benefits of particular choices’ (there, the risk was one associated with a particular-sized aortic valve replacement^136^). In *Webster*, the patient’s ‘background (a university degree in nursing)’ was referred to as one of the factors that indicated that the patient should have been told of the risks arising from delay in delivering a child with polyhydramnios. Both risks were considered to be material to *those patients* and should have been disclosed. In neither case did the factor operate alone, and it will never be conclusive, but case law suggests that an HCP should be cognizant, should a patient be able to particularly appreciate risk information because of their educational background.

##### e. The HCP’s knowledge of the patient’s behaviour, which behaviour is likely to place the patient at greater risk

Patients are not perfect creatures. They miss appointments, fail to take prescribed medication, and choose not to conduct the exercises which are recommended. Where an HCP knows of their patient’s less-than-careful behaviour, then any inherent risk that would be significantly heightened for that patient as compared with the general population may assume materiality. This factor may appear to be overly burdensome for an HCP when confronted with an uncooperative patient, but the factor is nevertheless relevant. For example, in *Thorp v Mehta*,^137^ where the patient, 42, died of a stroke, her estate sued her GP for failing to immediately prescribe antihypertensive drugs to control her high blood pressure, and for persisting with further diagnostic assessment instead. The GP knew that the patient had a patchy attendance record, and had previously failed to attend hospital and doctors’ appointments relating to the treatment of her blood pressure. The court was satisfied that a reasonable patient would likely have attached significance to the inherent risk of further delay in achieving a diagnostic assessment (and that the option of prescribing antihypertensives *pro tempore* should have been put to her).

##### f. The patient’s previous experiences of procedures and surgery

Where the patient has had previous treatment or surgery, then this should form part of an HCP’s reflection when advising of the inherent risks of a forthcoming treatment, for case law suggests that this can matter in *two* respects. First, if the patient’s prior surgery did not resolve the same medical problem *or* an inherent risk manifested with particularly unpleasant ramifications, then any risk that the proposed procedure could cause or exacerbate that same problem would matter to that particular patient. This was significant in *Biggadike v El Farra*,^138^ where the patient underwent a colposuspension procedure to treat urinary incontinence from which she had suffered since the birth of her second child. The procedure carried the risk of further complications (including that it could cause incontinence itself), which should have been discussed with the patient prior to the procedure. The risk was material, given that the patient was ‘very unwell’, and had already experienced two major operations which had not achieved the correction of incontinence that she had been hoping for. Secondly, if a patient had benefited from conservative treatment previously, then any risks associated with more interventionist treatment may be treated at law as being material for that reason. In *Hassell v Hillingdon Hospitals*,^139^ the patient had undergone osteopathy for lower back pain previously and had benefited from it; so that ‘surgery with a risk of paralysis would have been a frightening prospect’.^140^ The risk of paralysis should have been disclosed, bearing the patient’s previous experiences in mind.

##### g. How risk-taking or risk-averse was the patient

If the patient exhibits, in his answers or behaviour, a tendency to be *very* risk-averse, then this can interact with other already-mentioned factors to render a risk material in the legal sense. For example, in *Mills*,^141^ the patient lacked the capacity to litigate after the severe stroke, which he suffered following the minimally invasive resection procedure as treatment for a brain tumour. His wife gave evidence (which was accepted) that Mr Mills was averse to running any unnecessary risks, even distrusting the risks which were run when giving blood, and that he would not have agreed to any surgical technique which was not ‘tried and tested’ for fear of being a ‘guinea pig’. This factor operated together with other, already-mentioned, factors (eg, the risk was not present in a reasonable treatment option, and the procedure was very novel and rarely utilized amongst the profession of neurosurgery at that time).

##### h. The effect of the risk on the personal or family life of the patient

In *Montgomery* itself, the UKSC said that materiality could depend, inter alia, on ‘the effect which its occurrence would have upon the life of the patient’.^142^ It is a widely cast factor that has regard to the subjective characteristics of the particular patient and can cover a myriad of scenarios, rendering it quite a ‘dangerous’ factor for HCPs. It focuses attention on matters such as the patient’s family and marital circumstances, hobbies, and age. These are questions to which consultation should be directed for this reason. In *Thefaut v Johnston*,^143^ it was explicitly suggested that the impact of a risk’s manifestation on a patient’s hobbies or pastimes could render a risk material. Certainly, the impact of the risk of paralysis arising from spinal cord surgery on the family life of the patient mattered in *Hassell*,^144^ given that she was only 41, a mother of three children, and in full-time work as head of year at her school. In *Kennedy v Frankel* too,^145^ the risk of impulse control disorder and compulsive behaviour associated with dopamine agonist medication, and which could give rise to uncontrollable spending, was a material risk for the patient, ‘an intelligent woman’ who was married to a retired neurosurgeon, and for both of whom the patient’s behavioural changes were significant.

##### i. Intensely personal matters, such as fertility, cultural, or religious beliefs

The ability to bear children can constitute such an important matter of personal autonomy that any failure to warn that a risk of treatment might impede the ability to carry a future pregnancy may be material. Similarly, any risk which would produce an outcome that would be unacceptable for the patient on religious or cultural grounds is likely to be material too. In *Diamond*,^146^ where the patient complained that she was not informed that a mesh repair of a hernia could compromise any future pregnancy, that risk should have been warned about for that reason (but, ultimately, causation failed). In *Keh* too,^147^ where the patient was a Jehovah’s Witness, the risk of an emergency Caesarean section if the proposed NVD did not proceed smoothly meant that there was a potentially higher risk of blood loss (and Mrs Keh would not accept a transfusion). That was one factor that contributed to the finding that the patient was at significantly higher risk of danger if an emergency procedure was required, and that risk was material and should have been disclosed (however, again, causation failed).

##### j. The patient’s subjective willingness to run risks

This is a factor that pertains to the particular patient (ie the subjective limb of *Montgomery*), and operates on the basis that some patients will regard a risk as *not* being material because of their subjective views or characteristics, whereas a patient drawn from the general population may consider that risk to be very material. In other words, some post-*Montgomery* case law demonstrates that an HCP is entitled to take into account where the particular patient is willing to run risks. Some judicial examples are that a risk of serious and permanent pain may be less material to a patient who has a heightened tolerance for, or stoicism towards, pain, or has a greater ability to manage pain^148^; or that a mother might place great personal value on giving birth by NVD rather than by Caesarean section, and is prepared to assume the risks to herself and her baby which that decision would entail^149^; or a patient might have ‘volunteered his complete trust in his medical professionals’, especially if he had undergone similar successful procedures with those professionals previously.^150^ These are merely examples; the key point is whether anything indicates that the particular patient was prepared to *tolerate* material risks.

##### k. Any emotional fragility in the patient’s circumstances

In *Thefaut v Johnston*,^151^ it was suggested in dicta that a risk may be considered to be material (hence, disclosable) if the emotional capacity of the patient to take on further risk at that stage of their lives was very limited. Green J gave examples of where the patient ‘has suffered a recent event in his/her life (such as a bereavement or a divorce) which renders that person unusually fragile and (say) unwilling to take chances at that particular time’.^152^ The author’s searches have not found a real-life application of this factor to date, but the mere fact that it has been judicially suggested renders it worth noting in the map of the *Montgomery* Materiality Test.

##### l. A patient’s incessantly asking questions about particular outcomes or risks

In *Montgomery*, the UKSC remarked that ‘[e]xpressions of concern by the patient, as well as specific questions, are plainly relevant’ to the question of materiality. What the patient wants to know matters, *legally* as well as medically. Post-*Montgomery*, the patient’s failure to ask any questions about risks of surgery, which she later complained were not warned about, counted against materiality^153^; whereas conversely, the fact that the patient asked several questions about one risk (a hoarse voice) rendered it material.^154^ There is still probably no better case that illustrates this factor than the Australian case of *Rogers v Whitaker*,^155^ upon whose test the UKSC modelled theirs in *Montgomery*. The patient lost sight in her good eye, post-surgery, due to the manifestation of the risk of sympathetic ophthalmia. That extremely rare risk was material, partly because both prior to and on the day of surgery, Mrs Whitaker had ‘incessantly questioned [the surgeon] as to, amongst other things, possible complications. … On the day before the operation, Mrs Whitaker asked [the surgeon] whether something could be put over her good eye to ensure that nothing happened to it’.^156^ The ‘right question’ may not have been asked (how could it be, when even many textbook authors did not refer to the risk at the time), but the frequent questioning hallmarked the remote risk as being material.

### B. A guidance note and a practice note

The whole point of *Montgomery* is that the patient should be placed in the position whereby he ‘understands the seriousness of his condition, and the anticipated benefits and risks of the proposed treatment and any reasonable alternatives, so that he is then in a position to make an informed decision’.^157^ Putting the patient in that position, however, imposes a considerable burden upon the relevant HCP—and it is the appeasement of that burden to which this article is directed.

The aforementioned 20 factors derived from extensive case law analysis are reproduced in Appendix A as a Guidance Note, as a sort of directional ‘map’. It is hoped that (whilst not constituting legal advice) the Note will provide coherent, useful and practical assistance ‘at the coalface’, to assist HCPs (and their professional advisers and organizations) to appreciate what the *Montgomery* Materiality Test actually requires of them as they seek to navigate the post-*Montgomery* landscape. The Guidance Note does not (and, indeed, cannot) seek to dictate what the Materiality Test may look like a further decade from now; judicial attitudes will evolve, and disparate fact situations may arise, both of which will render ‘materiality’ a ‘living test’, and will require that it be kept under constant review. But 10 years on, it represents a guide for ‘*Montgomery* compliance’.

Moreover, on a practical level, courts have emphasized that HCPs should take proactive steps to provide patients with an information leaflet about risks while the patient awaits the procedure, and about which ‘meaningful opportunity’^158^ is provided to ask questions in a further consultation. The anticipatory time period is important, to avoid falling foul of *Montgomery’*s insistence that informed consent is not met ‘by routinely demanding her signature on a consent form’, especially on the day of surgery.^159^ It is best to avoid the ‘mutual momentum toward surgery’, as the court put it in *Thefaut v Johnston*.^160^ Failure to provide any risk literature ahead of time will count against the defendant HCP.^161^ To that end, a Practice Note to assist HCPs is set out in Appendix B.

## IV. Conclusion

The court put it well in *Powell v University Hospitals Sussex*: ‘[w]e live in an age where patient autonomy is taken very seriously. Gone are the days where we as patients are passive canvasses upon which medical practitioners develop and practice their skills.’^162^ But equally, Lady Hale’s extra-curial comment is entirely apposite, that *Montgomery* ‘has no doubt caused practical problems’.^163^ However, by undertaking a comprehensive analysis of post-*Montgomery* jurisprudence, this article has sought to provide legally cogent and practical assistance as to what the post-*Montgomery* landscape means for HCPs and for their legal representatives. This has been tackled in two ways.

First, it is essential to understand when a defendant HCP’s conduct will be judged against peer professional opinion per the *Bolam/Bolitho* test, and when it will be for the court to decide whether that HCP’s conduct sat on the ‘right’ side of what the law requires. The position waxes and wanes throughout the ‘consultation phase’, which occurs between diagnosis and treatment, courtesy of appellate rulings in *Duce* and *McCulloch*. It is a nuanced process, for even where *Bolam* applies, the court can instate its own view by virtue of the *Bolitho* gloss; and even where the court is required to be the final arbiter of risk disclosure per *Montgomery*, *Bolam* evidence may still be relevant.

Secondly, the *Montgomery* Materiality Test needs to be applied in medical consultations each and every day, up and down the country, and it is important that HCPs actually understand what it requires. Only a thorough analysis of case law, delivered in the decade since, can truly elucidate that, an analysis which has been undertaken herein. The materiality factors are reproduced as a suggested Guidance Note in Appendix A. These are divided into the objectively—and the subjectively—significant risks, in line with the division outlined by the UKSC in the *Montgomery* Materiality Test, albeit that the dividing line between the two will be blurred in some factual scenarios.

It has been judicially said that ‘[t]he obligation of the medics to inform is not and can never be limitless’.^164^ That is surely true–but the obligation upon HCPs, post-*Montgomery*, is undoubtedly onerous. Nevertheless, the law should *always* be capable of being understood and applied by those who are governed by it. A decade on from *Montgomery*, the analysis herein will hopefully enable HCPs and their legal advisors to achieve precisely that, in order to discharge the duty which the case truly envisioned.

## Supplementary Material

fwag012_Supplementary_Data

## Footnotes

1 A risk that cannot be eliminated by the exercise of reasonable skill and care, and which arises ‘from rare and random causes in every surgical procedure’: *Rogers v Whitaker* (1992) 175 CLR 479, [96].

2 [2015] UKSC 11, [2015] AC 1430.

3 Per the test in *Bolam v Friern Hospital Management Committee* [1957] 1 WLR 582 (QB) (‘*Bolam*’).

4 [2015] UKSC 11, [87].

5 ibid [68], but without any freestanding claim to damages merely because of some infringement of the patient’s personal autonomy in not receiving adequate information, eg: *Shaw v Kovac* [2017] EWCA Civ 1028, [65]; *Diamond v Royal Devon & Exeter Hospitals* [2019] EWCA Civ 585, [11], [35].

6 [1985] AC 871 (HL).

7 (1992) 175 CLR 479 (HCA).

8 [2015] UKSC 11, under the heading ‘comparative law’ at [70]ff.

9 See, eg, Lord Reed has pointed out, extra-curially, that in *Donoghue v Stevenson* [1932] AC 562 (HL), Lord Atkin cast his eye across the Atlantic to Cardozo J’s judgment in *MacPherson v Buick Motor Co* (1916) 217 NY 382 (Ct App, 1916), which recognized that a duty of care was not dependent upon privity of contract: ‘The Immortal Snail’ (Law Society of Scotland, *Donoghue v Stevenson* 90th Anniversary Conference, 26 May 2022).

10 See, eg: Mulheron, *Principles of Tort Law* (2nd edn, CUP 2020) 130–134 (‘Until *Montgomery*, both “reasonable patient” and *Bolam* tests had received strong appellate endorsement, and first-instance application, in English law’, citing numerous authorities).

11 See also, eg, *Birch v UCL Hospital* [2008] EWHC 2237 (QB), where the *Bolam* test supported non-disclosure of the risk of stroke from an angiogram, but the reasonable patient test required disclosure.

12 Quoted verbatim in *Montgomery* at: [2015] UKSC 11, [25] and [101].

13 ibid [94].

14 ibid [89].

15 Such as the Medical Defence Union, the General Medical Council, the British Dental Association, and other professional organizations that draft pro forma risks literature for their members.

16 See: *CNZ v Royal Bath Hospitals* [2023] EWHC 19 (KB) [264].

17 [2015] EWHC 3135 (QB) [108].

18
*Singh v Hospital Authority* [2020] HKCFI 530, [43]–[44].

19
*Prasad v Labasa Hospital* [2025] FJHC 130, extensively citing *Montgomery*.

20
*Law v Marura Maternity and Nursing Home* [2022] KEHC 17132, especially [182]–[184].

21
*Lee Kong Ghee v Andrew Khoo Kian Ming* [2023] SGDC 17, especially [51].

22 [2015] UKSC 11, [81] (‘The social and legal developments which we have mentioned point away from a model of the relationship between the doctor and the patient based on medical paternalism’).

23
*Thorp v Mehta* [2024] EWHC 652 (KB) [55].

24 [1998] AC 232 (HL) 243 (Lord Browne-Wilkinson).

25 This depends upon proof of one or more ‘*Bolitho* factors’ which have been examined by the author elsewhere: Mulheron (n 10) 368–375, which expanded upon and updated earlier analysis: ‘Trumping *Bolam*: A Critical Legal Analysis of *Bolitho’s* “Gloss”’ (2010) 69 Cambridge Law Journal 609. Nevertheless, some do not consider *Bolitho* to protect patients sufficiently from negligent medical judgment, eg: M Leitch, ‘Disclosing Alternative Treatments: Towards a *Bolam-Montgomery* Partnership’ (2024) 40 Professional Negligence 107, 111; L Sutherland KC, ‘The rebirth of medical paternalism?’ (2023) 175 Reparation Bulletin 3, 6.

26 See, eg, *Maynard v West Midlands Regional HA* [1984] 1 WLR 634 (HL) 638, 648; *Sidaway* [1985] 1 All ER 643 (HL) 659; and *Bolitho* [1998] AC 232 (HL) 241. The point was reiterated by the lower court in *McCulloch* and cited by the UKSC in *McCulloch* without adverse comment: [2023] UKSC 26, [35].

27 J McQuater, ‘*McCulloch* – Case Comment’ [2023] Journal of Personal Injury Law C215, C218.

28 See: *Butler v Ward* (KB, 10 Apr 2025) [41] (‘[t]he decision in *McCulloch* does change the approach to informed consent in relation to risks’).

29 [2018] EWCA Civ 1307.

30 [2023] UKSC 26, [2024] AC 925.

31 See, eg, *Tosh v Gupta* [2025] EWHC 2025 (KB) [4], [140]; *Adamson v Baillie County Court* (Newcastle CC, 24 Apr 2024) [8], [12]; *Robinson v Liverpool University Hospital* [2023] EWHC 21 (KB) [53]; *Johnstone v NHS Grampian* [2019] CSOH 90, [11].

32 Specifically, the consent ‘is not regarded as a nullity’, so that the claim cannot be brought in battery in the absence of fraud or bad faith, but only in negligence: *Shaw v Kovac* [2017] EWCA Civ 1028, [68], citing: *Chatterton v Gerson* [1981] QB 432, 443.

33 Acknowledged in *McCulloch* [2023] UKSC 26, [62].

34
*McCulloch* [2023] UKSC 26, [65]–[66].

35 Per: *Bayley v George Eliot Hospital* [2017] EWHC 3398 (QB) [99] (no breach in failing to advise patient that DVT could be treated by ilio-femoral venous stent; a reasonably competent vascular surgeon would not have known about the availability or potential use of this treatment in 2008).

36 [2023] UKSC 26, [57] (also termed, ‘clinically suitable’ alternative treatments, [59]).

37 ibid [58].

38 ibid [56]–[57].

39 ibid [56], by reference to the defendant cardiologist’s expert witness’s evidence (noted at [31]).

40 ibid [35].

41 See, eg: *Winterbotham v Shahrak* [2024] EWHC 2633 (KB) [67] (patient should have been told of coronectomy as a reasonable alternative treatment); *Hassell v Hillingdon Hospitals* [2018] EWHC 164 (QB) [77] (physio should have been offered as an option to spinal surgery); *CNZ v Royal Bath Hospitals* [2023] EWHC 19 (KB) [297]–[300] (patient should have been offered Caesarean section or artificial rupture of membranes).

42 [2023] EWCA Civ 605, [66] (Nicola Davies LJ wrote the judgment of the court).

43 [2018] EWCA Civ 1307, [33].

44 Noted ibid [25] and [44].

45 ibid [42].

46 Quoting E Cave and C Milo, ‘Informing Patients: The *Bolam* Legacy’ (2020) 20 Medical Law International 103.

47 As discussed by the author in: ‘Twelve Tests to Identify Whether a Material Risk is “Material”’ (2000) 1 National Law Review 1, [64]–[67], citing relevant cases in which *Bolam* evidence was described as ‘a useful guide’, ‘significant’, ‘of enormous assistance’, and ‘helpful’.

48 [2018] EWCA Civ 1307, [33] (emphasis added).

49 KB, 10 Apr 2025.

50
*Mills* [2019] EWHC 936 (QB) [193].

51
*Montgomery* [2015] UKSC 11, [85].

52 ibid [91].

53 ibid, [88].

54 R Mulheron, ‘Has *Montgomery* Administered the Last Rites to Therapeutic Privilege? A Diagnosis and a Prognosis’ [2017] 70 Current Legal Problems 149–188.

55 C Hobson, ‘Still no(,) more *Bolam* please’ (2024) 87 Modern Law Review 1586, 1599 (‘normatively, the paternalistic implications of applying the professional-practice [*Bolam*] test to the assessment of reasonable alternatives is problematic’); and R Heywood, ‘Medical disclosure of alternative treatments’ (2009) 68 Cambridge Law Journal 30, 31.

56 S Jaffer and R Reed-Berendt, ‘Defining the Boundaries of *Montgomery*: A Scottish Approach’ (2022) 38 Professional Negligence 105, 111.

57 Leitch (n 25) 114.

58 R Weir KC and T Westwell, ‘*Bolam* Returns by the Back Door’ [2023] Journal of Personal Injury Law 231, 231, citing: *Britten v Tayside Health Board* [2016] SC Dun 75, [23] (Sheriff Collins QC).

59 R Heywood and J Miola, ‘Back to the Future? Identifying an Appropriate Legal Approach to Judging Disclosure of Alternative Medical Treatments’ [2025] King’s Law Journal 23.

60 K Amirthalingam, ‘Medical Duty to Advise, Patient Autonomy and Reasonable Alternatives’ (2024) 140 Law Quarterly Review 11, 13.

61 Sutherland (n 25) 3.

62 This is the ‘overriding duty’ of the expert, per Civil Procedure Rules, r 35.3(1).

63 See eg, questions of fact; and matters falling outside of expert judgment, such as discharge-of-patient communications between medical staff, and managing the workload of on-call medical staff, lie outside the *Bolam/Bolitho* framework, as discussed in: Mulheron (n 10) 376–381.

64 See, eg: Leitch (n 25); Sutherland (n 25); Heywood and Miola (n 59).

65 As the author has discussed elsewhere: see (n 25).

66 The Inner Division noted that most of the expert evidence adduced in the court below was about *the process of the labour*, that is, the trace, its interpretation, etc: [2013] CSIH 3, [9]; while in the Outer House, the obstetrician’s expert evidence did not address the specific question as to whether a Caesarean was a reasonable option for Mrs Montgomery: [2010] CSOH 104, [70]–[73].

67 [2023] UKSC 26, [62].

68 See, eg, the perceptive discussion in Heywood and Miola (n 59).

69 See the discussion and authorities in source at (n 63).

70 K Liddell and others, ‘Differentiating Negligent Standards of Care in Diagnosis’ (2022) 30 Medical Law Review 33, 44, citing: *Muller v King’s College Hospital* [2017] EWHC 128 (QB, Kerr J). See too: Cave and Milo (n 46) (‘[t]here is a role for clinical expertise in the selection of relevant treatment options and in support of patients when they choose between them that is potentially disrupted by emphasis in *Montgomery* on consumerism’).

71 [2000] EWCA Civ 3035, [30], [43], [59], and discussed further by the author at (n 10) 366–367.

72 [2023] UKSC 26, [81].

73 See (n 26).

74 [2023] UKSC 26, [71], citing: *AH v Greater Glasgow Health Board* [2018] CSOH 57, [42]–[43].

75 Per *R (Burke) v GMC* [2006] QB 273 (CA) [50(v)].

76
*Rex (JJ) v Spectrum Community Health* [2023] EWCA Civ 885, [60] (citing *McCulloch*), [69], [73]; *Johnstone v NHS Grampian* [2019] CSOH 90, [150].

77 Adverted to in *Burke* (n 75), [50(v)].

78 J MacLeod, ‘Double Duty’ (2024) 28 Edinburgh Law Review 130, 133.

79 Cave and Milo (n 46), although these authors cite evidence that HCPs are under pressure ‘to comply with patient demands even when the options sought by patients are not clinically indicated’.

80 Amirthalingam (n 60) 15.

81 See text accompanying (n 4).

82 A view expressed elsewhere by the author in: *Principles of Tort Law* (n 10) 130.

83
*Butler v Ward* (KB, 10 Apr 2025) [33].

84
*Thefaut v Johnston* [2017] EWHC 497 (QB) [55].

85 ibid [54].

86 See, eg: *Di Carlo v Dubois* [2004] QCA 150, [77] (patient irrationally convinced he had a brain tumour, and suffered extreme anxiety; heightened risk of allergic anaphylactic reaction to non-ionic contrast agent not warned about; duty to warn of subjectively significant risk upheld).

87 [2015] UKSC 11, [89] (‘the nature of the risk, the effect which its occurrence would have upon the life of the patient, the importance to the patient of the benefits sought to be achieved by the treatment, the alternatives available, and the risks involved in those alternatives’).

88 Drawn from England, Wales, Scotland, and Northern Ireland, via a review of Bailii and Westlaw UK databases. Several of the sets did not address materiality; hence, this section refers to the subset that did.

89 [1985] AC 871 (HL), especially at 890.

90 (1998) 48 BMLR 118.

91 [2015] UKSC 11, [2015] AC 1430, [94].

92
*Plant v El-Amir* [2020] EWHC 2902 (QB) [82] (quote) and [17] (patient had age-related macular degeneration which was worse in her left eye, but present in her right eye too).

93
*Hassell* [2018] EWHC 164 (QB) [66].

94 To recall (per Section IA), quantification of risk is a matter of clinical judgment.

95 [2015] UKSC 11, [89].

96
*Thorp v Mehta* [2024] EWHC 652 (KB) [66].

97 As in *Ollosson v Lee* [156] (‘the word “small” is clearly an everyday word which encompasses and satisfactorily conveys the level of risk involved’).

98 e.g., *Spencer v Hillingdon Hospital* [2015] EWHC 1058 (QB) (no duty to warn of deep vein thrombosis or pulmonary embolism pre-operation; both described as ‘rare’ risks).

99
*Clements v Imperial College Healthcare* [2018] EWHC 2064 (QB) [56].

100 See, eg, the 9–10 per cent risk of shoulder dystocia in *Montgomery*; and see too: *Winterbotham v Shahrak* [2024] EWHC 2633 (KB) [66] (‘Mrs Winterbotham should have been told that her extraction was a high risk one … and that it carried a 2% risk of permanent nerve injury, and a 20% risk of temporary injury’); *Keh v Homerton University Hospitals* [2019] EWHC 548 (QB) (risk of emergency Caesarean section >15 per cent).

101
*Hassell* [2018] EWHC 164 (QB) [73].

102
*Tasmin v Barts Health* [2015] EWHC 3135 (QB) [115].

103 Section IA, Stage 1.

104 [2019] EWCA Civ 585, [4]. Only causation was appealed; and not the finding of materiality reached at first instance: [2017] EWHC 1495 (QB) [28].

105 [2015] UKSC 11, especially at [89], [94].

106 [2019] EWHC 936 (QB) (‘*Mills*’).

107 ibid [197], [201].

108 ibid [214]–[215].

109 Mulheron, ‘Duties in Contract and Tort’ in J McHale and J Laing (eds), *Principles of Medical Law* (5th edn, OUP 2025) [3.17]–[3.24].

110 [2015] UKHL 11, [81].

111 [1985] AC 871 (HL) 902.

112
*Norney v Watt* [2024] NIKB 78, [57], [61].

113
*Plant v El-Amir* [2020] EWHC 2902 (QB) [20], [82].

114
*Duce* [2018] EWCA Civ 1307 (dicta only; the risk of such pain was unknown at the time; hence, a *Montgomery* duty did not arise).

115
*Plant v El-Amir* [2020] EWHC 2902 (QB) [82].

116
*Thefaut v Johnston* [2017] EWHC 497 (QB) [78].

117 See, especially, the view of the Court of Appeal on this point: [2002] EWCA Civ 724, [5], [17] (Sir Denys Henry). On further appeal, only causation was in issue.

118 See Section IA.

119 [2005] 1 AC 134 (HL).

120 [2019] EWHC 784 (QB) [145].

121
*LT v Lothian Health Bd* [2019] CSIH 20, [35]; *Tasmin v Barts Health* [2015] EWHC 3135 (QB) [32], [108].

122 B de Boer and O Kudina, ‘What Is Morally at Stake When Using Algorithms to Make Medical Diagnoses?’ (2021) 42 Theoretical Medicine and Bioethics 245, 251.

123 Outlined at: <https://www.ibm.com/products/watsonx> (accessed 13 May 2025).

124 T Grote and P Berens, ‘On the Ethics of Algorithmic Decision-making in Healthcare’ (2020) 46 Journal of Medical Ethics 205, 207.

125
*Winterbotham v Shahrak* [2024] EWHC 2633 (KB) [62].

126
*Hassell* [2018] EWHC 164 (QB) [69] (that risk manifested, but so too did the undisclosed risk of paralysis).

127
*Butler v Ward* (KB, 10 April 2025).

128 [2015] UKSC 11, [7], [112].

129 [2019] EWHC 548 (QB) [64], [89].

130 [2021] EWHC 643 (QB) no pp.

131
*NKX v Barts Health* [2020] EWHC 828 (QB).

132
*McNab v Greater Glasgow Health Bd* 2020 CSOH 53, [107] (emphasis added).

133 ibid [122].

134 [2021] EWHC 643 (QB).

135 [2017] EWCA Civ 62, [41]. In this author’s view, *Webster* was correctly decided re the *Montgomery* claim, and respectfully disagrees with the view that it was wrongly decided: R Weir KC and T Westwell, ‘*Bolam* Returns by the Back Door’ [2023] Journal of Personal Injury Law 231, 235–236.

136 [2021] EWHC 643 (QB).

137 [2024] EWHC 62 (KB) [58].

138 [2024] EWHC 1688 (KB) [380].

139 [2018] EWHC 164 (QB) [66].

140 ibid, quote at [77].

141 [2019] EWHC 936 (QB) [208].

142 [2015] UKSC 11, [89].

143 [2017] EWHC 497 (QB) [56].

144 [2018] EWHC 164 (QB) [69].

145 [2019] EWHC 106 (QB) [49].

146 [2017] EWHC 1495 (QB).

147 [2019] EWHC 548 (QB).

148
*Thefaut v Johnston* [2017] EWHC 497 (QB) [55] (a dicta example).

149
*Webster v Burton Hospitals* [2017] EWCA Civ 62, [31] (another dicta example).

150 See, eg, *Inglis v Brand* [2016] SC Edin 63, [18] (lower wisdom tooth extraction left patient with numbness and tingling of tongue and jaw; risk not material; patient had undergone four previous extractions, and ‘volunteered his trust in medical professionals at the outset of his evidence’).

151 [2017] EWHC 497 (QB).

152 ibid [55].

153
*Tosh v Gupta* [2025] EWHC 2025 (KB) [142].

154
*Hassell* [2018] EWHC 164 (QB) [69] (and it was disclosed).

155 [1992] HCA 58, (1992) 175 CLR 479.

156 ibid 491.

157 [2015] UKSC 11, [90].

158
*Winterbotham v Shahrak* [2024] EWHC 2633 (KB) [62]–[64] (risks information handed over in silence).

159
*Hassell* [2018] EWHC 164 (QB) [74] (warning of spinal cord damage on day of operation not sufficient).

160 [2017] EWHC 497, [78].

161
*Norney v Watt* [2024] NIKB 78, [57], [61].

162 [2023] EWHC 736 (KB) [17].

163 Speech to the Legal Action Group, *Legal Aid at 70* (5 April 2019).

164
*C v Colchester Hospital* (CC Southend, 22 Feb 2018) [57]. [13,107]

## APPENDIX A

**Table:**

A GUIDANCE NOTE – MATERIALITY OF RISK, POST-*MONTGOMERY*	
When assessing the materiality of an inherent risk of a medical treatment or procedure, a risk is likely to be considered to be material, and hence disclosable, where one or more of the following **20** factors apply: ***The objectively-oriented factors:*** **1.** The resulting injury would be grave, life-changing, and permanent; **2.** The probability (or magnitude) of the risk could be measured in single digits (and even lower, if other factors apply); **3.** An alternative reasonable treatment option did not carry the same inherent risk; **4.** The treatment or procedure which gave rise to the risk was novel, innovative, and largely untested; **5.** The risk is an obvious risk, but its occurrence is of very high magnitude (greater than the balance of probabilities) or would cause grave physical consequences for the patient; **6.** The risk arises out of elective surgery, and demonstrates one of the other factors; **7.** The risk is one that a responsible body of peer medical opinion would regard as material and which those peers of the HCP would ordinarily disclose to their patients; **8.** The risk that information which underpins a recommended treatment is incorrect, irrelevant, or both. ***The subjectively-oriented factors:*** **9.** The resulting injury would hinder or preclude the patient from pursuing the ability to make a living in their existing or prospective occupation or profession; **10.** The patient’s own physical characteristics or existing medical condition elevates the probability of the risk compared with a patient without those characteristics or condition;	**11.** The patient’s medical history elevates the probability of the risk compared with a patient without that medical history; **12.** The patient’s educational background heightens the significance of the risk for that particular patient compared with the appreciation of that risk which would be held by a patient without that expertise; **13.** The HCP possesses knowledge of the behaviour of the patient which behaviour elevates the probability of the risk compared with a patient without that behaviour; **14.** The patient’s own previous experiences of medical treatment or procedures renders the risk more significant than for a patient without those previous experiences; **15.** The patient exhibits generally risk-averse behaviour which would render the risk significant to that particular patient; **16.** The manifestation of the risk would affect the personal or family life of the patient in a way that causes severe embarrassment, decreased quality of life, or inability to care for family members; **17.** The resulting injury would impact upon intensely personal matters such as the patient’s fertility, or cultural or religious beliefs; **18.** The patient is not willing to run that risk on a subjective basis (and, obversely, a risk is not material if that patient considers the risk to be less important than would a patient from the general population); **19.** The resulting injury would greatly impact the patient due to a life event that elevates the emotional fragility of the patient compared with other patients without that life event; **20.** The patient incessantly asked questions about the risk or of any adverse outcome resulting from that risk.

## APPENDIX B

**Table:**

**A PRACTICE NOTE – COMPLYING WITH THE *MONTGOMERY* DUTY**
When seeking to comply with the *Montgomery* Materiality Test, the following practice points may assist (derived from case law): Provide leaflets which contain risks information about the medical procedure in question; and provide the written literature at the earliest opportunity; Discuss verbally the risks contained in the leaflet at some time prior to (or even on the day of) the medical procedure; Explicitly ask the patient whether they have any questions associated with the risks; Make (and retain) brief contemporaneous notes of the risks disclosure conversation with the patient; this will assist the court to resolve any credibility dispute as to what was said which requires ‘axioms of fact-finding’; De-jargonise risks information to ensure that the message is conveyed in a comprehensible manner; and avoid bombarding the patient with highly technical information.

