# Supplementary material for: Ten years on: a ‘Montgomery map’ for healthcare professionals in the United Kingdom and Beyond
Source: Med Law Rev. 2026 Jun 11;34(2):fwag012. doi: 10.1093/medlaw/fwag012 (PMC13256225; doi:10.1093/medlaw/fwag012)
Supplement: fwag012_Supplementary_Data [file fwag012_supplementary_data.docx]

***APPENDIX A***

| **A GUIDANCE NOTE – MATERIALITY OF RISK, POST-*MONTGOMERY*** | |
| --- | --- |
| When assessing the materiality of an inherent risk of a medical treatment or procedure, a risk is likely to be considered to be material, and hence disclosable, where one or more of the following **20** factors apply:  ***The objectively-oriented factors:***  **1.** The resulting injury would be grave, life-changing, and permanent;  **2.** The probability (or magnitude) of the risk could be measured in single digits (and even lower, if other factors apply);  **3.** An alternative reasonable treatment option did not carry the same inherent risk;  **4.** The treatment or procedure which gave rise to the risk was novel, innovative, and largely untested;  **5.** The risk is an obvious risk, but its occurrence is of very high magnitude (greater than the balance of probabilities) or would cause grave physical consequences for the patient;  **6.** The risk arises out of elective surgery, and demonstrates one of the other factors;  **7.** The risk is one that a responsible body of peer medical opinion would regard as material and which those peers of the HCP would ordinarily disclose to their patients;  **8.** The risk that information which underpins a recommended treatment is incorrect, irrelevant, or both.  ***The subjectively-oriented factors:***  **9.** The resulting injury would hinder or preclude the patient from pursuing the ability to make a living in their existing or prospective occupation or profession;  **10.** The patient’s own physical characteristics or existing medical condition elevates the probability of the risk compared with a patient without those characteristics or condition; | **11.** The patient’s medical history elevates the probability of the risk compared with a patient without that medical history;  **12.** The patient’s educational background heightens the significance of the risk for that particular patient compared with the appreciation of that risk which would be held by a patient without that expertise;  **13.** The HCP possesses knowledge of the behaviour of the patient which behaviour elevates the probability of the risk compared with a patient without that behaviour;  **14.** The patient’s own previous experiences of medical treatment or procedures renders the risk more significant than for a patient without those previous experiences;  **15.** The patient exhibits generally risk-averse behaviour which would render the risk significant to that particular patient;  **16.**  The manifestation of the risk would affect the personal or family life of the patient in a way that causes severe embarrassment, decreased quality of life, or inability to care for family members;  **17.** The resulting injury would impact upon intensely personal matters such as the patient’s fertility, or cultural or religious beliefs;  **18.** The patient is not willing to run that risk on a subjective basis (and, obversely, a risk is not material if that patient considers the risk to be less important than would a patient from the general population);  **19.** The resulting injury would greatly impact the patient due to a life event that elevates the emotional fragility of the patient compared with other patients without that life event;  **20.** The patient incessantly asked questions about the risk or of any adverse outcome resulting from that risk. |

***APPENDIX B***

| **A PRACTICE NOTE – COMPLYING WITH THE *MONTGOMERY* DUTY** |
| --- |
| When seeking to comply with the *Montgomery* Materiality Test, the following practice points may assist (derived from case law):   1. Provide leaflets which contain risks information about the medical procedure in question; and provide the written literature at the earliest opportunity; 2. Discuss verbally the risks contained in the leaflet at some time prior to (or even on the day of) the medical procedure; 3. Explicitly ask the patient whether they have any questions associated with the risks; 4. Make (and retain) brief contemporaneous notes of the risks disclosure conversation with the patient; this will assist the court to resolve any credibility dispute as to what was said which requires ‘axioms of fact-finding’; 5. De-jargonise risks information to ensure that the message is conveyed in a comprehensible manner; and avoid bombarding the patient with highly technical information. |
